# Multidimensional regulation of estrogen signaling in pelvic floor connective tissue homeostasis and remodeling

**DOI:** 10.3389/fimmu.2026.1742246

**Published:** 2026-01-22

**Authors:** Lin Wang, Mengyu Geng, Lingyun Wei, Shuyu Wang, Wenzhen Wang, Xiaochun Liu

**Affiliations:** 1 Third Hospital of Shanxi Medical University, Shanxi Bethune Hospital, Shanxi Academy of Medical Sciences, Tongji Shanxi Hospital, Taiyuan, China; 2 Shanxi Bethune Hospital, Shanxi Academy of Medical Sciences, Third Hospital of Shanxi Medical University, Tongji Shanxi Hospital, Taiyuan, China

**Keywords:** cellular senescence, estrogen receptor, extracellular matrix, mechanotransduction, pelvic organ prolapse, signaling pathway

## Abstract

Pelvic organ prolapse (POP) is a prevalent condition that significantly impairs women’s quality of life and is closely linked to dysregulated estrogen signaling. This review examines the mechanisms through which estrogen, acting via nuclear receptors (ERα and ERβ) and the membrane receptor G protein-coupled estrogen receptor (GPER), regulates pelvic floor connective tissue homeostasis through both genomic and non-genomic pathways. Key regulatory effects include the promotion of collagen and elastin synthesis, inhibition of matrix metalloproteinase (MMP) activity, modulation of fibroblast function—including mitigation of cellular senescence and enhancement of migratory and anti-apoptotic capacities—as well as integration with mechanical signaling through the integrin-YAP/TAZ axis. Additionally, estrogen helps suppress chronic inflammation and influences macrophage polarization. Clinical evidence indicates that estrogen deficiency and an elevated ERα/ERβ ratio contribute to extracellular matrix degradation, a hallmark of POP. Although local estrogen therapy can improve perioperative tissue quality, its long-term efficacy in structural restoration remains limited. Emerging therapeutic approaches include selective estrogen receptor modulators, ERβ-specific agonists, and personalized interventions based on receptor profiling and genetic markers. Future research should leverage patient-derived organoid models and targeted drug delivery platforms to decipher individual pathophysiology and translate findings into precise interventions.

## 1 Introduction

Pelvic organ prolapse (POP) represents a major disorder of pelvic floor dysfunction, posing a significant and growing global public health challenge (1). Epidemiological studies indicate that up to 50% of women may demonstrate anatomical evidence of POP on examination (1), with about 11–19% undergoing surgical intervention during their lifetime (2). By 2050, the number of cases is projected to increase by 46%, reaching approximately 156 million worldwide (3). This condition not only imposes a substantial socioeconomic burden, driven significantly by the direct and indirect costs of surgical management, but also severely impairs quality of life, manifesting as urinary dysfunction, dyspareunia, recurrent infections, and reduced psychosocial well-being (1, 4).

The integrity of pelvic support structures relies on a dynamic balance between connective tissue composition, cellular function, and mechanical adaptation (5–7). Central to this balance is the extracellular matrix (ECM)—primarily type I/III collagen and elastic fibers (6)—whose homeostasis is critically influenced by hormonal signaling. Among these regulators, estrogen has emerged as a key modulator of pelvic floor tissue remodeling, yet its role remains complex and context-dependent.

Although estrogen deficiency in postmenopausal women is strongly correlated with increased POP risk (8), the hormone’s effects are not uniformly protective. Pregnancy-related high-estrogen states, for example, coincide with ligament laxity (9), and clinical outcomes of estrogen therapy are often inconsistent (10). This ambiguity points to a multifaceted regulatory system involving not only circulating hormone levels but also receptor subtype expression (ERα, ERβ, and GPER) (11–13), genomic versus non-genomic signaling, and cross-talk with inflammatory and mechanotransduction pathways.

Current understanding of estrogen’s role in POP is fragmented. While basic research highlights its ability to promote collagen synthesis, inhibit matrix degradation, and modulate fibroblast behavior (14, 15), clinical translation has been limited by heterogeneous patient responses, variability in receptor profiles (11–13), and a lack of individualized therapeutic strategies (16–18). Moreover, emerging dimensions such as cellular senescence, immune microenvironment remodeling, and estrogen-mechanical signaling integration have yet to be systematically incorporated into a cohesive pathogenic model.

Therefore, this review aims to: (1) synthesize current evidence on the multidimensional mechanisms of estrogen signaling in pelvic floor connective tissue homeostasis; (2) delineate how dysregulation of these pathways contributes to POP pathogenesis; (3) evaluate existing and emerging estrogen-based therapies, with emphasis on precision medicine approaches; and (4) identify key research gaps and future directions for translating mechanistic insights into improved clinical outcomes.

The following sections will systematically explore the molecular mechanisms of estrogen action, its integrative role in tissue remodeling, the pathological alterations in POP, and the translational potential of targeted hormonal interventions.

## 2 Molecular mechanisms of estrogen signaling pathways

### 2.1 Receptor-mediated genomic effects (classical pathway)

The genomic effects mediated by estrogen receptors represent the classical pathway of estrogen signal transduction, involving sophisticated regulation by the nuclear receptor superfamily members ERα and ERβ. Although these receptor subtypes share a highly conserved DNA-binding domain (approximately 95% homology), they exhibit significant differences in tissue distribution and biological functions. In female pelvic floor support tissues, ERα is predominantly expressed in the uterine and vaginal epithelium, whereas ERβ is more abundant in fibroblasts and smooth muscle cells of pelvic floor connective tissue (19). This distribution pattern aligns with clinical observations: ERα knockout mice exhibit infertility due to impaired uterine response to estrogen (20), whereas ERβ-deficient mice, despite reduced fertility, maintain normal uterine and vaginal morphology, suggesting a distinct role for ERβ in preserving mechanical integrity of the pelvic floor (21). The molecular cascade of receptor activation begins with estrogen diffusion into the cell and specific binding to the ligand-binding domain (LBD), inducing a conformational change that exposes the nuclear localization signal. ERα and ERβ can form homo- or heterodimers, with αα dimers demonstrating significantly higher affinity for classical estrogen response elements (EREs) than ββ dimers (22). This differential binding affinity may underlie the tissue-specific responses observed in estrogen signaling.

The ligand-receptor complex binds directly to estrogen response elements (EREs, 5′-GGTCAnnnTGACC-3′) in the promoter regions of target genes via zinc finger motifs, or indirectly regulates gene expression through transcription factors such as AP-1 and Sp1. It is noteworthy that the promoters of type I/III collagen genes contain non-canonical EREs, whose regulation depends on the interaction between ERβ and Smad3 (23). The bidirectional nature of transcriptional regulation is reflected in the dynamic recruitment of coregulators: the estrogen-ER complex typically recruits coactivators such as SRC-1 and AIB1, forming a transcription initiation complex with histone acetyltransferase activity (24). In contrast, selective estrogen receptor modulators (SERMs) like tamoxifen tend to recruit corepressors such as NCoR and SMRT, suppressing transcription through histone deacetylation (25). This “molecular switch” mechanism is particularly critical in pelvic floor tissues.

**Figure 1:**
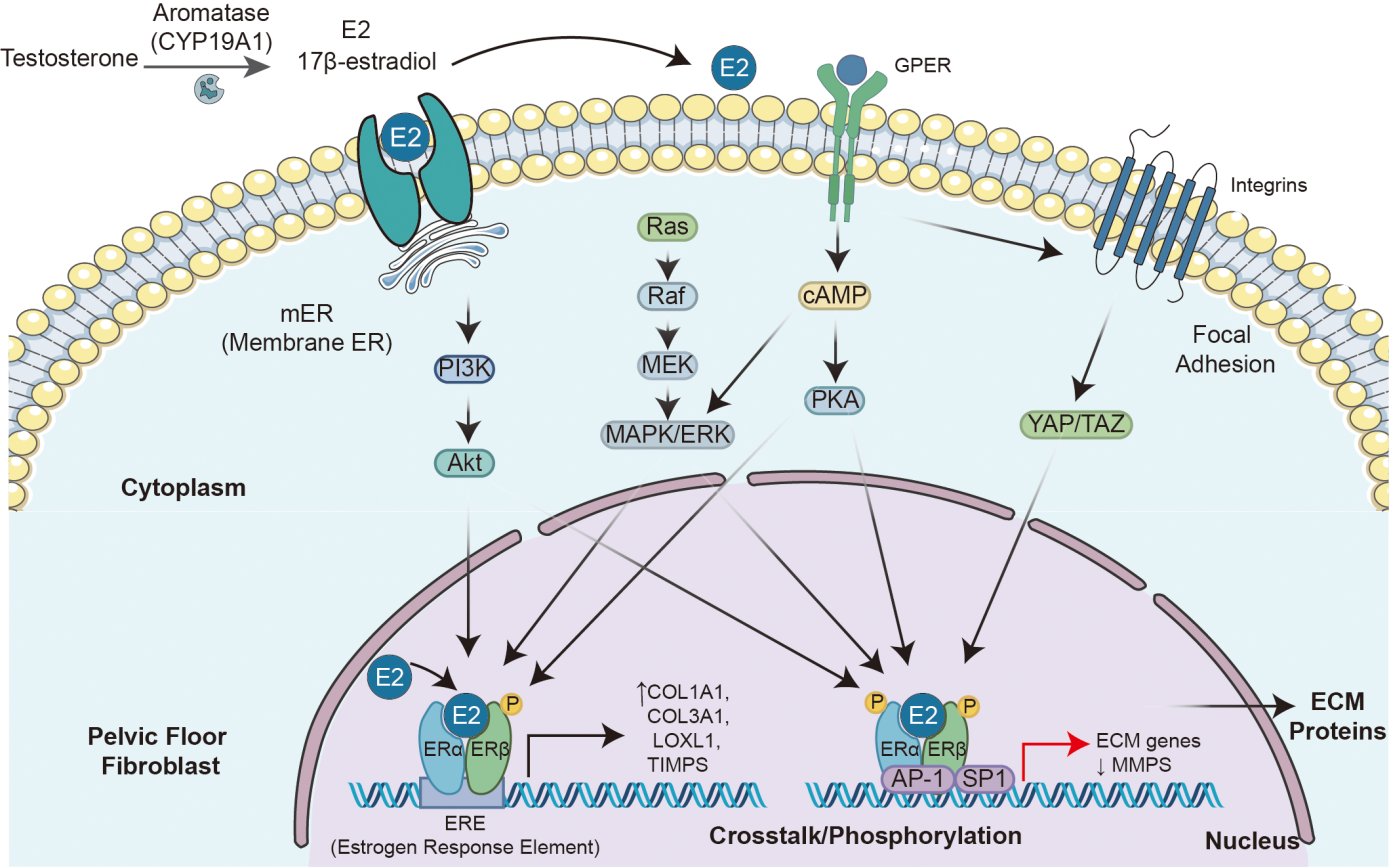
Integrated regulatory mechanisms of estrogen signaling in pelvic floor fibroblasts.

In the regulation of target genes associated with connective tissue remodeling, estrogen upregulates the expression of TIMP-1 and TIMP-2 directly through ERα, while simultaneously suppressing the transcription of MMP-2 and MMP-9 via the ERβ/AP-1 pathway (26). This dual regulatory mechanism becomes impaired in patients with pelvic organ prolapse, characterized by reduced ERβ binding and increased c-Fos/c-Jun binding at the MMP-9 promoter region (27). Estrogen enhances vaginal wall repair capacity by markedly upregulating LOX expression and promoting collagen cross-linking. In menopausal models (e.g., ovariectomized animals), estrogen not only increases elastic fiber density in the vaginal wall but also sustains LOX gene activation and elevates the proportion of mature collagen, thereby optimizing the long-term matrix microenvironment for wound healing (28). Notably, HOXA13—a key transcription factor regulating ECM components (e.g., collagen, fibronectin)—shows significantly reduced expression (14-fold decrease) in vaginal tissues from POP patients. This reduction is independent of estrogen status (unaffected by menopause or leuprolide treatment), revealing the existence of an estrogen-independent regulatory pathway for ECM metabolism (29). More importantly, clinical tissue analyses demonstrate a significantly elevated ERα/ERβ ratio in the pelvic connective tissues (e.g., vesicovaginal and rectovaginal septum) of POP patients. Premenopausal patients exhibit a 2.4-fold increase in ERα expression accompanied by decreased ERβ, while postmenopausal patients show an ERα/ERβ ratio 2.7 times higher than that of healthy controls (29.6 vs. 10.8) (12). These findings indicate that predominant ERα expression is a central feature of disrupted connective tissue homeostasis. Collectively, these insights provide a new perspective on the molecular mechanisms of POP: not only decreased estrogen levels, but also dysregulation of the ERα/ERβ expression ratio may be a critical factor undermining the stability of connective tissue (**Figure 1**).

### 2.2 Non-genomic effects (rapid signaling pathways)

Estrogen regulates gene transcription not only through classical genomic pathways but also rapidly activates intracellular signaling via non-genomic effects. This process is typically mediated by membrane-associated estrogen receptors, such as GPER1 (GPR30), and involves multiple kinase cascade reactions. As a G protein-coupled receptor, GPER1 is primarily localized to the endoplasmic reticulum and mediates rapid estrogen signaling at the plasma membrane. Upon activation, it triggers downstream effectors including phospholipase C (PLC), protein kinase C (PKC), and calcium signaling, thereby modulating cellular functions. This receptor binds estrogen with high affinity (Ki ≈ 6 nM) as well as certain anti-estrogen drugs, such as tamoxifen and ICI 182,780 (30). As a key receptor mediating broad physiological and pathological effects of estrogen—spanning cancer progression, cardiovascular function, metabolic homeostasis, inflammatory response, and neuroprotection—GPER exhibits complex pharmacological characteristics. Its ligands include endogenous estrogen, various pharmaceutical agents (e.g., tamoxifen, fulvestrant), and natural/synthetic environmental estrogens (e.g., plant isoflavones, bisphenols). Selective agonists (e.g., G-1, LNS8801) and antagonists (e.g., G15, G36) have been developed as research tools and potential therapeutic agents (31). In metabolic regulation, GPER1 coordinates energy and glucose homeostasis through dual pathways: on one hand, it enhances insulin secretion and survival in pancreatic β-cells via the cAMP/PKA and PLC/IP3 pathways (32); on the other hand, it promotes mitochondrial biogenesis and fatty acid oxidation in adipose tissue via the ERK/PI3K-Akt signaling axis, while simultaneously inhibiting adipocyte differentiation and the release of inflammatory cytokines (33). Activation of these signaling pathways can affect cell migration, proliferation, and survival within minutes.

Another key mechanism of estrogen’s non-genomic effects involves the activation of intracellular kinase networks through membrane-associated ERα or ERβ. For instance, upon binding to membrane receptors, estrogen can activate the PI3K-AKT-mTOR signaling pathway within minutes (34). Additionally, estrogen activates pathways such as ERK1/2, which function in various cell types. For example, in breast cancer cells, estrogen activates ERK1/2 via GPER1 (GPR30), regulating biological processes including cell proliferation and migration (35). The activation of these rapid signaling pathways enables estrogen to influence cellular functions within a short time frame, independent of gene transcription and protein synthesis. Although these rapid signaling events do not directly alter gene expression, they can indirectly modulate genomic effects by regulating the phosphorylation status of transcription factors such as c-Fos or CREB, thereby creating “crosstalk” with genomic signaling pathways. Specifically, estrogen binding to membrane-localized GPER1 or ERα variants rapidly activates MAPK pathways (e.g., ERK1/2). The activated MAPK subsequently phosphorylates and activates transcription factors, facilitating their nuclear translocation and ultimately regulating the transcription of specific genes, including those encoding miRNAs (36).

The integration of genomic and non-genomic effects is a central feature of estrogen signaling. Estrogen not only regulates gene expression through nuclear receptors (genomic effects) but also rapidly activates intracellular signaling via membrane receptors (e.g., PI3K/Akt), leading to eNOS activation and NO release (non-genomic effects). These two pathways interact bidirectionally—rapid signaling can modulate transcription, while transcriptional products support rapid cellular responses—and act synergistically to mediate key physiological and pathophysiological effects of estrogen on endothelial cells (37). This bidirectional crosstalk is particularly critical in tissue remodeling. For example, during wound healing, estrogen promotes fibroblast migration through rapid ERK activation while concurrently upregulating collagen synthesis via genomic actions, thereby synergistically enhancing tissue repair (38).

### 2.3 Estrogen metabolism and the local tissue microenvironment

When exploring the interplay between estrogen metabolism and the local tissue microenvironment, particular attention should be given to the regulation of local aromatase expression and activity, which controls the potential for estrogen synthesis within tissues. Aromatase (CYP19A1), the rate-limiting enzyme in estrogen biosynthesis, converts androstenedione and testosterone into estrone (E1) and estradiol (E2), respectively. Reduced aromatase expression in tissues may lead to insufficient local estrogen synthesis, thereby affecting collagen metabolism (39). In postmenopausal women, the decline in ovarian function is accompanied by a significant reduction in aromatase activity, resulting in decreased local estrogen levels (40).

Within the local tissue microenvironment, estrogen metabolism and receptor expression exert a critical influence on tissue function. For instance, the use of aromatase inhibitors (such as anastrozole and letrozole) in postmenopausal women significantly reduces serum estradiol levels, thereby affecting bone metabolism (41). It is noteworthy that the regulation of aromatase varies across tissues. For example, in endometriotic lesions, inflammatory cytokines such as IL-6 and TNF-α can markedly upregulate aromatase expression via activation of the NF-κB pathway (42). This tissue-specific regulation may explain differences in the local estrogen microenvironment between the pelvic floor tissues of POP patients and those with hormone-dependent disorders such as endometriosis. Furthermore, studies have shown that estrogen helps maintain the elasticity and strength of connective tissue by regulating collagen synthesis and cross-linking (43). The decline in aromatase activity in postmenopausal women is associated with reduced collagen cross-linking, suggesting that diminished estrogen synthesis capacity may contribute to connective tissue degeneration.

The differential bioactivity of estrogen metabolites represents another critical dimension. Beyond classical estradiol (E_2_), estrone (E_1_) and estriol (E_3_) can also bind to the estrogen receptor (ER) with varying affinities. Studies indicate that estradiol (E_2_) serves as the natural ligand for both ERα and ERβ, exhibiting the highest binding affinity for these receptors (44). More notably, while E_2_ is the most biologically active form of estrogen (e.g., promoting proliferation), its metabolites such as 2-hydroxyestradiol (2OH-E_2_) and 4-hydroxyestradiol (4OH-E_2_) may exhibit markedly different activities, including anti-proliferative and pro-apoptotic effects. These metabolites can form DNA adducts, thereby inducing DNA damage and mutations, and contributing to carcinogenic processes (45). Furthermore, the balance between sulfatase and sulfotransferase activities regulates the storage and activation of estrogen sulfates. This regulatory process is significantly dysregulated in the vaginal epithelium of postmenopausal women, which may be associated with diminished local estrogen responsiveness (46).

Estrogen signaling often exhibits a complex biphasic regulatory characteristic in the inflammatory microenvironment. Studies have shown that estrogen activates PI3K, thereby inhibiting the translocation of NF-κB from the cytoplasm to the nucleus and suppressing its activation (47). Furthermore, in the pelvic floor tissues of patients with POP, local estrogen helps improve the structural integrity of postmenopausal pelvic floor support tissues by inhibiting the activity of inflammation-related proteases (such as MMP-9 and MMP-12) and promoting collagen synthesis (14). However, high concentrations of estrogen may carry pro-inflammatory and tissue-damaging risks (48). Particularly in pelvic floor tissues, systemic high-dose estrogen can induce phenotypic transformation of vascular smooth muscle cells (VSMCs), fostering a chronic inflammatory microenvironment that directly contributes to vascular pathology. Such vascular damage may indirectly compromise the stability of pelvic support structures, underscoring the importance of precise regulation of estrogen concentration for maintaining pelvic floor function (49).

At the molecular level, the crosstalk between estrogen signaling and inflammatory pathways involves multi-node regulation. Transforming growth factor-β1 (TGF-β1) acts as a central hub in this process. When estrogen levels decrease, dysregulation of the TGF-β1 signaling pathway—such as diminished suppression of the Smad pathway—can lead to disrupted ECM metabolism and contribute to the development of pelvic floor dysfunction (50). Notably, estrogen-mediated inhibition of MMP-2 transcription via the ERα/MAPK/Elk-1 signaling axis molecularly antagonizes the stimulatory effect of TGF-β1 on MMP-2 expression. Through their coordinated regulation of key nodes in ECM metabolic homeostasis, these two pathways form a bidirectional regulatory network that collectively maintains the structural integrity of pelvic support tissues (51).

## 3 Multidimensional regulatory mechanisms of estrogen in pelvic floor connective tissue remodeling

### 3.1 Regulation of ECM metabolic balance

#### 3.1.1 Promotion of collagen and elastin synthesis

Estrogen plays a key role in regulating ECM homeostasis. In POP, the connective tissues of the pelvic floor consistently exhibit impaired ECM metabolism, which is characterized by significantly reduced collagen content, disorganized collagen fibers, and elevated activity of matrix-degrading enzymes (52, 53). Specifically, the expression levels of type I and III collagen are significantly reduced in the uterosacral ligaments of POP patients compared to non-POP individuals (52). As these collagens are the main components of the pelvic support structure, their deficiency directly leads to weakened tissue support, thereby initiating or exacerbating POP (53).

To counteract this extracellular matrix deficit, estrogen promotes the synthesis of its key components. Specifically, as described in Section 2.1, estrogen enhances the synthesis of type I and III collagen in pelvic floor fibroblasts via the ERα-mediated genomic pathway (54). In a rhesus monkey model, estrogen treatment increased the gene expression of collagen I/III in pelvic support tissues (54). Research confirms that E2 significantly promotes the expression of type I and III collagen *in utero*sacral ligament fibroblasts from POP patients, supporting the view that estrogen regulates ECM balance by influencing collagen synthesis (55).

A key mechanism involves E2 activating the HOXA13/TIMP1 axis (56). HOXA13 is associated with estrogen receptors and morphological changes of collagen and elastic fibers in the uterosacral ligament, playing a role in POP development (56). TIMP1, as an endogenous inhibitor of MMPs, effectively inhibits collagen degradation. Therefore, by enhancing the HOXA13/TIMP1 axis, E2 not only promotes collagen synthesis but also reduces its degradation, thereby helping to restore pelvic floor ECM balance (56). Furthermore, E2 can promote the proliferation, migration, and collagen production of uterosacral ligament fibroblasts from POP patients (56, 57).

Beyond collagen, estrogen also modulates tissue elasticity by influencing elastin metabolism. Elastin, a key ECM component responsible for tissue resilience, is often dysregulated in POP. Research indicates that differential expression of the estrogen receptor GPR30 correlates with expression levels of elastin as well as type I and III collagen in pelvic support ligaments. This suggests that estrogen helps maintain the elasticity and mechanical strength of pelvic tissues by regulating the dynamic balance of these critical ECM components through receptor-mediated pathways such as GPR30 (58). This suggests that estrogen may affect the dynamic balance of these key ECM components through receptor-mediated signaling pathways such as GPR30, thereby maintaining the elasticity and strength of pelvic floor tissues (58). In addition, estrogen enhances the transcription of tropoelastin and fibronectin (59).

#### 3.1.2 Inhibition of matrix metalloproteinase activity

Estrogen is a central regulator that counteracts ECM degradation through a coordinated array of mechanisms targeting MMPs. A foundational mechanism is the direct transcriptional regulation of MMP genes. Research indicates that estrogen can inhibit the expression of the pro-enzymes of MMP-2 and MMP-9 in pelvic connective tissue fibroblasts, effectively reducing the available pool of these degradative enzymes (60). This aligns with broader findings demonstrating that estrogen treatment significantly suppresses the expression of MMP-2 and MMP-9. Beyond transcriptional control, estrogen also promotes the post-translational clearance of active MMPs; for example, it accelerates the fragmentation of MMP-13 via the ubiquitin-proteasome pathway, thereby directly removing existing enzymes from the ECM (61). Concurrently, estrogen upregulates the expression of endogenous inhibitors, the tissue inhibitors of metalloproteinases. It promotes the expression of TIMP-1 and TIMP-2, which inactivate MMPs by forming stable complexes (62). This upregulation of TIMPs establishes a critical biochemical counterbalance to MMP activity.

In addition to direct molecular regulation, estrogen modulates the broader signaling environment that controls MMP activity. It influences pathways responsible for converting MMP zymogens into their active forms (63). Moreover, estrogen exerts a systemic anti−inflammatory effect, suppressing pro−inflammatory cytokines such as TNF−α—key stimulators of MMP expression and activity—thereby indirectly mitigating ECM degradation in pelvic tissues. This anti−inflammatory role provides a crucial link to the dysregulated tissue microenvironment discussed in the following section (64).

The decline in estrogen levels, a hallmark of the postmenopausal state, critically disrupts this multifaceted regulatory network. The resultant imbalance, characterized by elevated MMP activity coupled with diminished TIMP expression, accelerates the catabolism of pelvic connective tissue ECM, constituting a central pathophysiological pathway in the development of POP. Beyond regulating degradation, estrogen is also crucial for enhancing ECM quality and structural maturation. In animal models, estrogen supplementation significantly increases the proportion of mature, cross-linked collagen in the vaginal wall, directly enhancing its ultimate biomechanical strength (28).

### 3.2 Regulation of cellular behaviors

#### 3.2.1 Regulation of cell proliferation, survival, and anti-apoptosis

In POP, dysfunctional fibroblasts within the compromised pelvic floor connective tissue are a key factor in disease progression. Estrogen, primarily as 17β−estradiol (E2), acts to preserve tissue homeostasis by exerting multifaceted regulatory effects on fibroblast proliferation, survival, and resistance to apoptosis (52, 65).

Estrogen promotes pelvic floor tissue integrity through an integrated control of fibroblast function. A fundamental action is the stimulation of fibroblast proliferation, which is essential for tissue repair and ECM renewal. Specifically, E2 enhances the proliferation of human uterosacral ligament fibroblasts (hUSLFs) derived from POP patients. This pro−proliferative effect is concentration−dependent, with significant enhancement observed at physiological E2 concentrations *in vitro* (55, 56). The underlying mechanism involves the synergistic action of classical genomic signaling via nuclear receptors (ERα/ERβ), which upregulates cell−cycle genes such as CCND1, and rapid non−genomic signaling initiated by membrane receptors like GPER1, which activates pathways including MAPK and PI3K−AKT (36). Furthermore, the activation of specific regulatory axes, such as HOXA13/TIMP1 by E2, links the promotion of collagen synthesis to the maintenance of a robust fibroblast population, forming the cellular foundation for sustained ECM production (56).

In addition to promoting cell proliferation, estrogen provides a crucial anti-apoptotic signal to maintain an adequate population of functional cells within the pelvic connective tissue. Research demonstrates that E2 can inhibit apoptosis in hUSLFs derived from POP patients, thereby significantly enhancing cell survival (55). This protective effect is evident under both basal conditions and in the context of mechanical stress, highlighting estrogen’s role in bolstering cellular resilience (55).

The significance of this function is underscored by the clinical observation that estrogen deficiency, particularly in postmenopausal women, constitutes a major risk factor for POP (65). This deficiency is hypothesized to shift the cellular equilibrium towards reduced proliferation and increased apoptosis, ultimately disrupting the delicate balance between ECM synthesis and degradation.

Within the dynamic mechanical environment of the pelvic floor, the adaptation of fibroblasts to sustained mechanical stress is essential for maintaining tissue function (66, 67). Studies show that mechanical stress alters hUSLFs morphology and function, activating stress-responsive pathways like p38 MAPK (66). Estrogen appears to modulate this response protectively. A key pathological event is the differentiation of fibroblasts into a more contractile, pro-fibrotic phenotype induced by a high-stiffness matrix microenvironment—a process implicated in POP. Estrogen can inhibit this stiffness-induced differentiation (68). This inhibition may be mediated through epigenetic mechanisms, such as the enhancement of DNA methyltransferase 1 (DNMT1) expression, which helps maintain genomic stability and preserves the fibroblast’s functional phenotype, preventing maladaptive changes (68). These regulatory actions are further refined at the epigenetic level, fine−tuning the expression of the pro−survival and pro−proliferative gene network orchestrated by estrogen.

The regulatory influence of estrogen extends into broader signaling networks. Dysregulation of estrogen signaling, often manifested as altered receptor expression, is closely associated with abnormal cellular behaviors in POP. Estrogen, through its receptors, orchestrates the expression of downstream genes that govern the cell cycle, survival, and differentiation. For example, *in vitro* evidence suggests that E2 may regulate fibroblast dynamics by modulating the expression of mitochondrial proteins like Mitofusin-2 (Mfn2) (52). Furthermore, the involvement of pathways such as Wnt signaling, mediated by receptors like FZD3 in regulating fibroblast viability and ECM metabolism, points to a complex signaling landscape where estrogen likely engages in extensive crosstalk (69). These actions are further refined by epigenetic modifications, including histone acetylation, which fine-tunes the expression of the pro-survival and pro-proliferative gene network orchestrated by estrogen (36).

Based on the above mechanistic insights, the estrogen regulatory network offers potential therapeutic targets and intervention strategies for pelvic floor functional restoration. Future translational research holds promise for breakthroughs in areas such as tissue-specific estrogen modulation, targeted intervention of key nodal pathways, and multimodal combination therapies.

#### 3.2.2 Regulation of cell migration and tissue repair

Cell migration is essential for tissue repair, enabling fibroblasts to reach injury sites and remodel the extracellular matrix. Damage to pelvic floor connective tissue—from factors such as childbirth or estrogen decline—can impair this migratory capacity, compromising intrinsic repair and contributing to POP development (65).

Estrogen, primarily in the form of 17β-estradiol, orchestrates fibroblast migration and tissue repair through multiple molecular pathways. A key action is the direct enhancement of cell motility: studies demonstrate that E2 significantly promotes the migratory capacity of human uterosacral ligament fibroblasts (hUSLFs) derived from POP patients (56). Effective migration is a critical prerequisite for fibroblasts to reach injury sites and secrete new extracellular matrix components, such as collagen and elastin, to facilitate tissue repair (56). Furthermore, by activating regulatory axes such as HOXA13/TIMP1, estrogen concurrently promotes collagen synthesis and indirectly supports cell migration (56). It also optimizes the repair microenvironment by modulating the balance between MMPs and TIMPs, thereby coordinately promoting directional cell migration and functional tissue regeneration (70).

At the intracellular level, estrogen influences the dynamic process of cell migration by modulating cytoskeletal remodeling. Cell movement requires precise reorganization of the actin cytoskeleton, which is governed by small GTPases of the Rho family (e.g., RhoA, Rac1, Cdc42). Estrogen is postulated to regulate the activity of these key signaling molecules, thereby influencing actin polymerization, stress fiber formation, and ultimately affecting fibroblast adhesion, spreading, and directional movement. Estrogen may also guide cell recruitment by regulating the expression of specific chemokines and their receptors on fibroblasts, enhancing their homing to damaged areas.

Concurrently, estrogen fosters a conducive local microenvironment for repair through its potent anti-inflammatory and antioxidant actions. While inflammation is a natural post-injury response, chronic or excessive inflammation impedes healing and can promote fibrosis (67). Estrogen mitigates this by suppressing the release of pro-inflammatory cytokines (e.g., TNF-α, IL-6) and promoting the production of anti-inflammatory mediators like IL-10, thereby attenuating detrimental inflammation and creating a favorable milieu for cell migration and ECM reconstruction (67). Additionally, estrogen helps modulate reactive oxygen species (ROS) levels, protecting cells from oxidative stress damage, which is crucial for maintaining cellular viability and function during repair (67). A key mechanism in preserving tissue architecture during this dynamic process is estrogen’s ability to upregulate E-cadherin (CDH1) expression upon binding to its nuclear receptors. This action stabilizes adherens junctions between epithelial cells, suppresses the epithelial-mesenchymal transition (EMT), and prevents abnormal cellular transdifferentiation, thereby maintaining structural integrity during remodeling (71).

#### 3.2.3 Anti-senescence effects and modulation of cell differentiation

Estrogen exerts potent anti−senescence effects on pelvic floor connective tissue. It directly upregulates collagen synthesis in fibroblasts, helps restore their contractile phenotype, and inhibits MMP−9−driven collagen degradation. By reducing tissue stiffness and attenuating mechano−induced α−SMA expression, estrogen helps break the “stiffness−fibrosis” cycle. Simultaneously, it remodels the TGF−β1/TIMP balance and promotes the timely resolution of the myofibroblast population, thereby delaying degenerative changes (70). In regulating differentiation, estrogen suppresses the cross−activation of the TGF−β1/Smad and RhoA/ROCK2 pathways, which prevents abnormal fibroblast differentiation into pathological myofibroblasts and instead promotes a functional, reparative phenotype that supports tissue homeostasis and regenerative capacity (72).

### 3.3 Regulation of the inflammatory and immune microenvironment

POP is closely associated with chronic inflammation and disruption of the local immune microenvironment (73). Tissues from POP patients, particularly vaginal tissues, frequently exhibit elevated inflammatory markers and increased infiltration of immune cells such as macrophages and lymphocytes, which actively drive pathological extracellular matrix remodeling and tissue degradation (73). Estrogen plays a key regulatory role in counteracting this inflammatory state. Estrogen exerts direct anti-inflammatory effects, such as by suppressing the nuclear translocation of NF-κB to downregulate pro-inflammatory cytokines like TNF-α and IL-6 (73, 74). It also modulates oxidative stress, potentially inhibiting the mechanical stress-induced reactive oxygen species (ROS) that activate the NLRP3 inflammasome—a key driver of pyroptosis and IL-1β release that exacerbates inflammation in POP (67).

Beyond suppressing inflammation, estrogen actively shapes a pro-reparative immune state. A central mechanism involves driving macrophage polarization away from the pro-inflammatory M1 phenotype and towards the anti-inflammatory, tissue-remodeling M2 phenotype (67). This shift, characterized by markers like CD206 and arginase-1, reorients macrophages from promoting ECM degradation to supporting its synthesis and constructive remodeling (75). Estrogen also regulates the activity and recruitment of other immune cells, including T and B lymphocytes, whose infiltration is associated with ECM alterations in POP (73). These actions occur through classical estrogen receptor (ERα/ERβ) signaling and involve cross-talk with other pathways, such as the Wnt signaling mediated by receptors like FZD3 (69, 76). Furthermore, estrogen may synergize with regenerative approaches, potentially enhancing the immunomodulatory capacity of adipose-derived mesenchymal stem cells (ADSCs), which are studied for their ability to stabilize the injury microenvironment and promote repair through paracrine signaling (67).

The integrated outcome of estrogen’s multifaceted regulation is the maintenance of immune homeostasis within pelvic tissues, ensuring that inflammatory responses are self−limiting and that the microenvironment supports ECM preservation and pelvic floor stability (66). Consequently, the decline in estrogen levels disrupts this balance, leading to a sustained pro−inflammatory, catabolic state that actively promotes POP progression (66, 67).

## 4 Dysregulation of estrogen signaling in the pathogenesis of pelvic organ prolapse

### 4.1 Estrogen deficiency and receptor imbalance as key drivers of POP

Epidemiological data consistently show a marked increase in POP incidence among postmenopausal women, strongly correlating with the sharp decline in circulating estrogen levels (1, 77). This correlation is underpinned by a direct biological link: estrogen deficiency leads to a reduction in total collagen content within pelvic floor tissues, a finding observed even when controlling for menopausal status (77). Interventional studies provide partial validation; a six-week preoperative course of local estrogen therapy can increase vaginal epithelial thickness, stimulate collagen synthesis, and inhibit MMP activity (14, 78). However, the relationship is not linear. Paradoxically, some studies indicate that combined estrogen-progestogen therapy may increase the risk of uterine prolapse, with risk escalating with longer treatment duration (79, 80). This contradiction highlights that the net biological effect of estrogen depends not only on its concentration but critically on the expression and balance of its receptors.

A central pathological feature in POP is the dysregulation of estrogen receptor (ER) subtype expression. Clinical tissue analyses reveal a significantly elevated ERα/ERβ ratio in the pelvic connective tissues (e.g., vesicovaginal and rectovaginal septum) of POP patients. Premenopausal patients exhibit a 2.4-fold increase in ERα expression coupled with decreased ERβ, while postmenopausal patients show an ERα/ERβ ratio 2.7 times higher than healthy controls (29.6 vs. 10.8) (12, 54). This imbalance disrupts the normal transcriptional programs regulated by these receptors. Since ERα homodimers have higher affinity for classical estrogen response elements (EREs) than ERββ dimers (22, 25), a predominant ERα expression may skew signaling towards a distinct set of target genes, potentially one that is less favorable for maintaining ECM homeostasis. Furthermore, the expression of the membrane receptor GPER is also altered in POP, showing significant upregulation in tissues like the uterine myometrium, where it may exacerbate abnormal ECM remodeling through non-genomic pathways (81–83).

### 4.2 The dysfunctional estrogen signaling network in POP and its downstream consequences

The imbalance in ER expression directly translates into aberrant transcriptional regulation of genes critical for extracellular matrix (ECM) metabolism. In pelvic floor tissues from POP patients, a characteristic “dual-hit” pattern is observed: the expression of key collagen synthesis genes (COL1A1, COL3A1) is downregulated, while the expression of matrix-degrading enzymes (MMP-2, MMP-9) is markedly upregulated (82, 84). This shift from an anabolic to a catabolic state is a molecular hallmark of POP. The impairment extends to enzymes responsible for ECM maturation; significantly reduced expression of lysyl oxidase (LOX) and its family members (LOXL1, LOXL3) is found in vaginal tissues from premenopausal POP women, a defect exacerbated by estrogen deficiency (85, 86).

At the level of intracellular signaling pathway activity, POP tissues exhibit characteristic abnormalities. The activities of the PI3K/Akt and MAPK/ERK pathways are altered, closely associated with the signaling bias resulting from imbalanced ER expression (87, 88). Under mechanical strain—a constant stress on pelvic floor tissues—dysregulated activation of the PI3K/Akt pathway may contribute to cellular damage rather than adaptive responses (88). Estrogen’s normal protective modulation of these kinase cascades is therefore compromised. Furthermore, the TGF-β1/Smad pathway, a key mediator downstream of estrogen for collagen production, shows reduced activity in POP, further exacerbating insufficient matrix synthesis (50, 89–91).

Local estrogen metabolism and concentration also become dysregulated in POP. Although circulating estrogen declines after menopause, local concentrations in pelvic floor tissues may show regional heterogeneity due to variations in the expression of aromatase (CYP19A1), the enzyme responsible for local estrogen synthesis (39, 75). More importantly, altered activity of estrogen-metabolizing enzymes (e.g., CYP1B1, COMT) may lead to the localized accumulation of bioactive estrogen metabolites. Some of these metabolites can induce oxidative stress and cellular damage via receptor-independent mechanisms, adding another layer of complexity to the pathology (80, 92).

### 4.3 Integrated model of pathological changes in pelvic floor connective tissue induced by aberrant estrogen signaling

The pathogenesis of pelvic organ prolapse can be understood through an integrated model in which dysregulated estrogen signaling acts as a central node, driving pelvic support failure via multifaceted mechanisms. The decline in estrogen levels or efficacy following menopause initiates a cascade of pathological events.

The loss of estrogen’s genomic regulation first disrupts the metabolic equilibrium of the extracellular matrix. Diminished activation of ERα and ERβ, combined with an unfavorable ERα/ERβ ratio, leads to reduced transcription of collagen and elastin genes and a failure to adequately suppress matrix metalloproteinase gene expression (15, 26, 62). The TGF-β1/Smad pathway, a crucial mediator of estrogen’s pro-fibrotic effects, becomes less active, further tipping the balance toward net matrix degradation (50, 90).

This dysregulation further extends to non-genomic and mechanotransduction pathways. Under normal conditions, estrogen enhances cellular responsiveness to mechanical loading by modulating integrins and the YAP/TAZ pathway, promoting adaptive ECM remodeling (93–95). In POP, this integrative function falters. Deficiencies in elastic fiber assembly proteins such as fibulin-5 (FBLN5) and LOXL1—whose expression is influenced by estrogen—result in poorly assembled, fragile elastic networks that critically impair tissue resilience (96–98). Clinical studies confirm significantly reduced levels of LOXL1 and elastin in the uterosacral ligaments of POP patients (82, 85).

Concurrently, the anti-inflammatory and immunomodulatory role of estrogen is lost. Postmenopausal estrogen deficiency permits the release of pro-inflammatory cytokines (e.g., TNF-α, IL-6), activation of the NF-κB pathway, and a shift in macrophage polarization toward the pro-inflammatory M1 phenotype (40, 47, 74). This chronic low-grade inflammatory microenvironment actively promotes ECM degradation. Furthermore, the overexpression of microRNAs such as miR-221/222 in POP patients can post-transcriptionally silence ERα, creating a vicious cycle that further diminishes functional estrogen signaling (96, 99).

**Figure 2:**
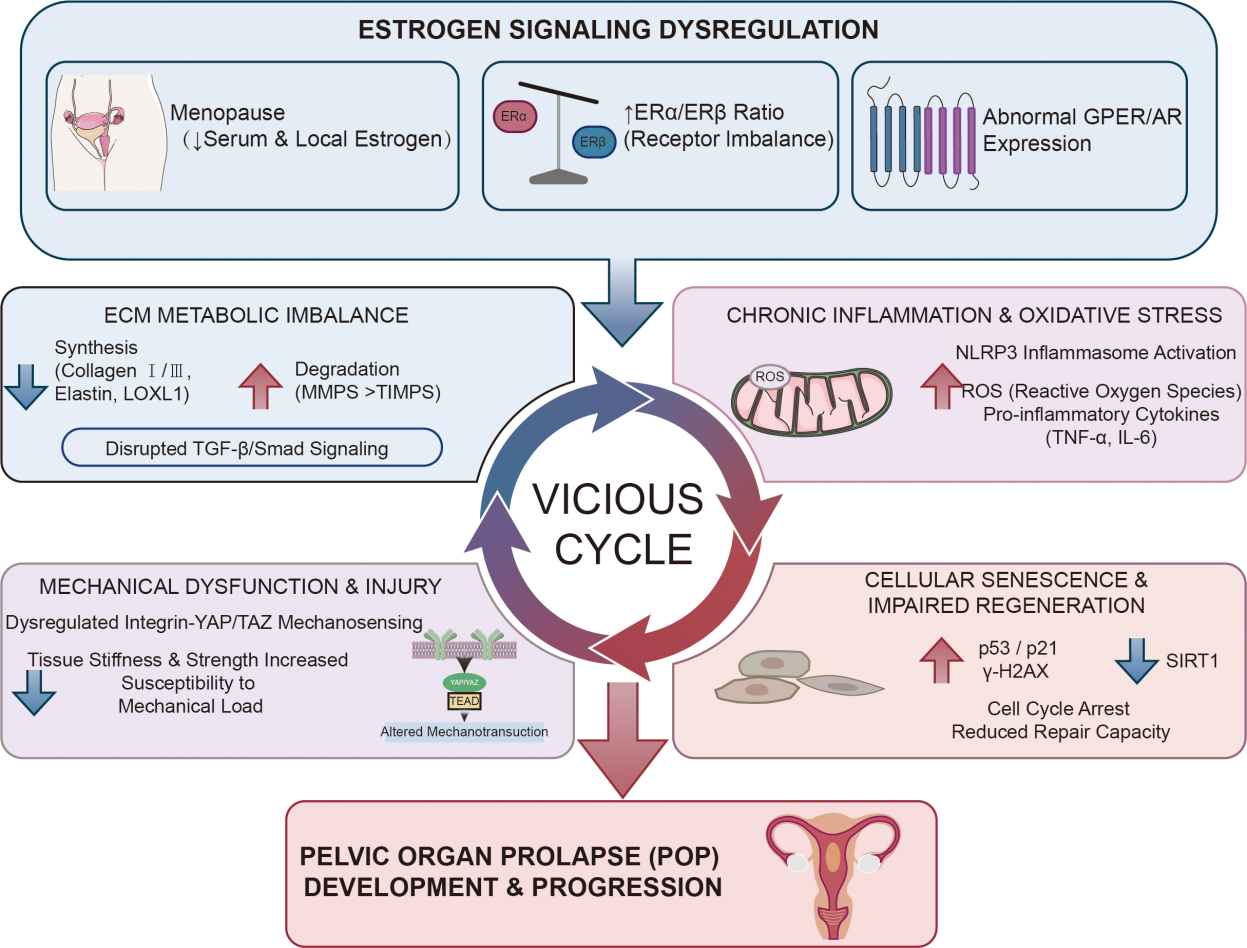
Integrated model of POP pathogenesis driven by aberrant estrogen signaling.

In summary, estrogen signaling dysregulation in POP—characterized by hormonal deficiency, receptor ratio imbalance, and disrupted downstream pathways—orchestrates a pathological program featuring reduced matrix synthesis, accelerated degradation, impaired mechanical adaptation, and chronic inflammation. This integrated failure of connective tissue homeostasis ultimately manifests as the structural weakness and functional incompetence of pelvic support structures that defines pelvic organ prolapse (**Figure 2**).

## 5 Targeted therapeutic strategies

### 5.1 Current estrogen-based therapies

#### 5.1.1 Local estrogen therapy

Local estrogen therapy, including vaginal creams, suppositories, or rings, has been widely utilized in postmenopausal women with POP, with clinical evidence partially supporting its efficacy (14). A randomized controlled trial demonstrated that six weeks of preoperative local estrogen treatment significantly increased vaginal epithelial and muscular layer thickness, promoted collagen synthesis, and suppressed the activity of degradative enzymes such as MMPs (14). The underlying mechanism may involve local activation of ERα/ERβ signaling, upregulation of collagen I/III transcription, and inhibition of MMP-2/9 activity (60). Preoperative application aims to improve vaginal tissue quality, theoretically providing a superior matrix for surgical suturing and reducing the risk of suture failure (14). Another study found that 2–12 weeks of preoperative estrogen treatment restored vaginal cytology to premenopausal levels, offering a more optimal anatomical plane for surgical manipulation despite no significant increase in epithelial thickness (100). Immediate postoperative initiation of a low-dose estrogen vaginal ring in postmenopausal women after vaginal reconstructive surgery was feasible and improved tissue quality during the healing period, reflected by higher vaginal maturation values and lower pH (101). However, histological improvements do not always translate into long-term clinical benefits; a randomized trial found that perioperative estrogen therapy did not significantly reduce prolapse recurrence rates at 3-year follow-up (102). Furthermore, regarding mesh-augmented surgery, preoperative vaginal estrogen treatment did not reduce the mesh exposure rate at one year postoperatively, suggesting it may not be routinely necessary in such cases (102). The efficacy of local estrogen appears limited in patients with severe structural prolapse, and long-term maintenance is often required (17). Importantly, some randomized controlled trials indicate that short-term (e.g., 6-week) vaginal estrogen application did not significantly improve patients’ subjective prolapse symptoms (10).

#### 5.1.2 Systemic hormone therapy

The role of systemic hormone therapy (e.g., oral estrogen) in POP management is more controversial and its effects are less clear compared to local therapy (14). Although estrogen may help maintain elastic fiber homeostasis in pelvic floor connective tissue (12), epidemiological studies suggest that systemic hormone replacement therapy (HRT) may increase the risk of uterine prolapse (80). This increased risk appears to be associated with longer treatment duration (79). The clinical outcomes of systemic estrogen replacement therapy (ERT) are contradictory, as some studies report no significant improvement in POP symptoms (10). Furthermore, the use of systemic HRT requires careful weighing of potential risks, including those for cardiovascular disease and breast cancer (103). The discrepancy between the clear regulatory effects of estrogen on ECM metabolism seen in basic research (104) and the lack of consistent symptomatic benefit in clinical studies highlights a significant translational gap. This may be related to the heterogeneity in study design, including variations in the route of administration, dosage, and treatment duration (17). Notably, long-term systemic estrogen therapy may be counterproductive by potentially activating MMPs and contributing to a chronic inflammatory microenvironment that could indirectly compromise pelvic support stability (49, 105).

#### 5.1.3 Selective estrogen receptor modulators

SERMs represent a promising therapeutic direction due to their tissue-specific effects, acting as agonists in some tissues (e.g., bone) while functioning as antagonists in others (e.g., breast) (16, 60). Preclinical studies have shown that raloxifene reduces MMP-2 activity in pelvic tissues of ovariectomized animal models (106). However, clinical evidence regarding their impact on POP is inconsistent and reveals a “tissue-selectivity paradox.” A retrospective study found that women over 60 years old treated with raloxifene for three years had a significantly reduced need for POP surgery (107). In contrast, other studies have reported that certain SERMs (e.g., levormeloxifene) may increase the risk of prolapse, potentially due to excessive activation of MMPs (108). This inconsistency underscores the significant heterogeneity in the effects of different SERMs on ECM metabolism. For instance, raloxifene demonstrates relatively weak MMP inhibitory effects compared to other agents (109). These pharmacological differences may explain why some SERMs are associated with an increased risk of pelvic floor dysfunction despite their beneficial effects on bone tissue (16, 108). The variable outcomes highlight the complexity of targeting estrogen receptors in a tissue-specific manner for POP and emphasize the need for more precise agents or better patient stratification.

### 5.2 Critical appraisal: risks, controversies, and limitations

#### 5.2.1 Safety profile and risks

The safety profile of estrogen-based therapies varies significantly between local and systemic administration routes. Local estrogen therapy (e.g., vaginal creams, tablets) is generally considered to have a favorable safety profile due to its low systemic absorption and targeted action (17). However, concerns regarding minimal systemic absorption and its potential effects, particularly on the endometrium, persist and warrant consideration (107). In contrast, the safety risks associated with systemic HRT are more substantial and well-documented. Long-term systemic estrogen therapy is associated with increased risks of endometrial pathologies, venous thromboembolism, and breast cancer, which significantly restrict its clinical applicability, especially for non-urogenital indications (103, 107). These systemic risks necessitate a careful risk-benefit assessment before initiation and highlight why systemic HRT is not recommended primarily for POP management (80, 103). The controversy surrounding systemic estrogen is further compounded by epidemiological data indicating that combined estrogen-progestogen therapy may actually increase the risk of uterine prolapse, with longer duration of use correlating with higher risk (79). Additionally, high concentrations of systemic estrogen may induce a pro-inflammatory phenotype in vascular smooth muscle cells, fostering a chronic inflammatory microenvironment that could indirectly undermine pelvic support (49).

#### 5.2.2 The efficacy paradox

A central paradox in estrogen therapy for POP is the frequent dissociation between demonstrable histological or biochemical improvement and meaningful long-term symptomatic relief or anatomical restoration. While preclinical and perioperative studies clearly show that estrogen improves vaginal epithelial thickness, promotes collagen synthesis, and inhibits MMP activity (14, 60), randomized controlled trials often fail to show significant improvement in patients’ subjective prolapse symptoms or long-term reduction in recurrence rates (10, 102). Several interrelated factors may explain this efficacy paradox. First, the duration of therapy may be critical; short-term interventions (e.g., 6 weeks) may only transiently inhibit degradation, whereas long-term stimulation (>3 months) is likely required for substantial *de novo* collagen synthesis and structural restoration (110). Second, in advanced POP, there may be irreversible structural damage and extensive ECM remodeling that exceeds the reparative capacity of estrogen monotherapy, suggesting a missed therapeutic window (65). Third, heterogeneity in patient populations and receptor expression influences response; for example, an elevated ERα/ERβ ratio or reduced ERα expression in pelvic tissues is associated with diminished sensitivity to estrogen therapy (12, 99). Finally, a mismatch between assessment endpoints plays a role. Traditional anatomical metrics (e.g., POP-Q stage) may not capture functional improvements better reflected in patient-reported outcomes (PROs), while histological improvements in tissue quality may not directly translate to measurable changes in support mechanics or symptom resolution (111). This paradox underscores the substantial translational gap between mechanistic understanding and clinical application (104).

#### 5.2.3 The tissue-selectivity paradox of SERMs

The tissue-selective actions of SERMs, while beneficial in certain contexts, create a distinct paradox in POP management. SERMs like raloxifene are designed to exert agonist effects in tissues such as bone (reducing fracture risk) while acting as antagonists in breast tissue (reducing cancer risk) (16). However, this selectivity does not extend uniformly to pelvic floor connective tissue, and their effects here can be neutral or even detrimental (108). The explanation for this paradox lies in the complex pharmacology of SERMs and tissue-specific receptor context. The net biological effect of a SERM in a given tissue depends on the local expression ratios of ERα and ERβ, the repertoire of available co-regulators (coactivators vs. corepressors), and the activation of distinct downstream signaling pathways (24, 25). In pelvic floor fibroblasts, certain SERMs may fail to adequately inhibit MMP activity or may even promote ECM degradation pathways, unlike their protective effects on bone matrix (106, 109). For instance, while raloxifene shows MMP-inhibitory effects in some models (106), its overall impact on pelvic floor ECM metabolism is considered relatively weak, and other SERMs like levormeloxifene have been associated with increased prolapse risk, potentially via excessive MMP activation (108). This highlights that the “selectivity” of SERMs is not binary and that a compound beneficial for bone may lack efficacy or be harmful for pelvic support structures due to fundamental differences in tissue microenvironment and estrogen receptor signaling dynamics (16, 108).

### 5.3 Optimizing perioperative application

The perioperative application of local estrogen is grounded in its ability to improve vaginal tissue quality, thereby theoretically optimizing surgical conditions and outcomes (14). Preoperative use, typically initiated 4–6 weeks before surgery, has been shown to significantly increase vaginal epithelial and muscular layer thickness, stimulate type I collagen synthesis, and suppress the activity of key MMP (14). These histological changes aim to provide a more robust and vascularized tissue plane for surgical dissection and suturing, potentially reducing intraoperative technical difficulty and the risk of suture pull-through (14, 100). A study specifically assessing the surgeon’s intraoperative perception of tissue quality found that preoperative estrogen treatment led to tissues being rated as less friable and easier to handle (112).

The potential benefits and optimal timing of postoperative application remain a subject of debate. Postoperative local estrogen may promote wound healing by increasing TGF-β1 expression and reducing inflammation and matrix degradation in the early recovery phase (113). Initiating a low-dose estrogen vaginal ring immediately after vaginal reconstructive surgery has been shown to be feasible and to improve tissue quality markers during healing (101). However, the timing of initiation may be critical. Animal studies suggest that immediate postoperative estrogen administration might be detrimental to matrix repair, possibly by disrupting the normal early inflammatory phase of healing, indicating a potential need for a delayed start (114). Furthermore, while estrogen can downregulate collagenase activity which is crucial for maintaining repair integrity (14), long-term clinical benefits such as reduced prolapse recurrence or mesh exposure rates have not been consistently demonstrated (102). For managing small, asymptomatic mesh exposures (<1 cm²), local estrogen therapy is recommended as a first-line conservative approach alongside observation (115).

Current clinical practice reveals significant heterogeneity in protocols and key knowledge gaps. While most studies employ a 4–6 week preoperative regimen, postoperative treatment duration varies widely from 3 to 12 months (14, 116). The choice of agent (conjugated estrogen cream, estradiol vaginal tablets, estriol preparations) also lacks standardization (112, 116). Major gaps include a lack of consensus on the optimal postoperative initiation timing and duration, insufficient evidence on the synergy between estrogen therapy and specific surgical techniques or biomaterials, and a need for precision protocols based on individual molecular biomarkers rather than a one-size-fits-all approach (114). The IMPROVE trial highlighted that perioperative estrogen did not significantly reduce prolapse recurrence, underscoring the gap between histological improvement and long-term clinical success (102).

### 5.4 Future directions and personalized medicine framework

#### 5.4.1 Novel pharmacological targets

Future therapeutic development is moving beyond conventional estrogen to target specific receptors and pathways. GPER-specific ligands (e.g., agonist G-1) are of interest given the upregulation of GPER in the uterosacral ligaments of POP patients and its role in mediating rapid, non-genomic signaling through pathways like PI3K/Akt (83, 117). Tissue-selective estrogen complexes (TSECs), which combine estrogens with a SERM like bazedoxifene, aim to provide the benefits of estrogen on vaginal tissue and bone while blocking unwanted stimulation of the endometrium, showing promise in animal models (118). Additionally, selective ERβ agonists are being explored as a strategy to potentially counteract the negative effects associated with a high ERα/ERβ ratio, promoting ECM synthesis and anti-inflammatory effects.

#### 5.4.2 Biomarker-guided personalization

A personalized medicine framework for POP requires stratification based on molecular profiling. Key proposed biomarkers include: 1) Estrogen receptor profiles: The ERα/ERβ expression ratio and GPER status in pelvic tissues could predict therapeutic response and guide the choice between ERα-targeting agents, ERβ agonists, or GPER modulators (12, 83, 99). 2) Genetic background: Polymorphisms in genes critical for ECM integrity (e.g., LOXL1, FBLN5) and estrogen metabolism (e.g., CYP1B1, COMT) or receptor genes (e.g., ESR1 rs2228480) may identify individuals with inherent connective tissue weakness or differential drug metabolism (15, 18, 92, 119). 3) Local inflammatory and metabolic markers: Profiles of MMPs, TIMPs, TGF-β, and advanced glycation end products (AGEs) could indicate the dominant pathological process (degradation, fibrosis, aging) and guide combination therapies (75, 120, 121).

#### 5.4.3 Technological innovations

Innovations in drug delivery and biomaterials are crucial to enhance efficacy and reduce side effects. Local sustained-release systems, such as estrogen-loaded nanoparticles or hydrogels, could prolong drug residence time in the vaginal tissue, provide more consistent dosing, and minimize systemic absorption (122). Advanced biodegradable and biocompatible scaffolds (e.g., electrospun poly-4-hydroxybutyrate) loaded with estrogen or bioactive molecules have shown potential in preclinical studies to support fibroblast infiltration, oriented tissue regeneration, and delay cellular senescence (123–125). These smart biomaterials can act as a temporary matrix while delivering therapeutic cues in a spatiotemporally controlled manner.

#### 5.4.4 Integrated management

**Figure 3:**
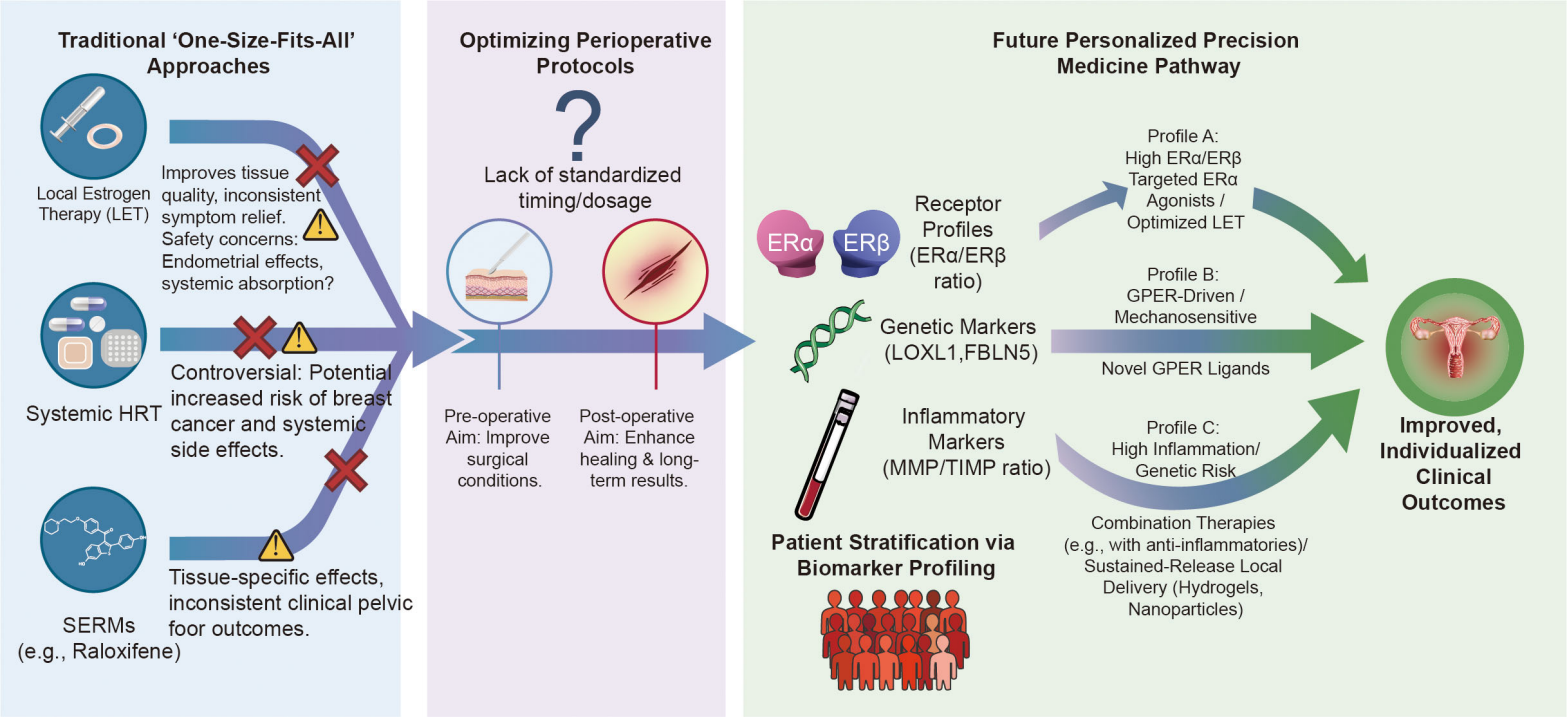
Evolution of therapeutic strategies and personalized decision pathway for estrogen signaling in POP.

Given the multifactorial etiology of POP, future management should explore integrated, multimodal strategies. Combining local estrogen therapy with pelvic floor muscle training (PFMT) may yield synergistic effects, as estrogen improves tissue quality and vascularity while PFMT enhances active muscular support; however, the mechanisms of synergy and long-term efficacy require robust clinical validation (126). Furthermore, for patients with comorbidities like diabetes, where AGEs accelerate tissue aging, combination therapy of estrogen with AGEs inhibitors (e.g., pyridoxamine) could be a strategic approach to address concurrent pathological pathways (120, 121). The integration of biomechanical assessments and advanced imaging (e.g., dynamic MRI, elastography) with molecular profiling will be key to developing truly personalized and comprehensive management plans (127) (**Figure 3**).

## 6 Research models and methodologies (foundations for mechanistic and translational research)

### 6.1 *In vitro* models

In the investigation of pathological mechanisms and treatment strategies for female POP, *in vitro* models serve as essential tools for understanding the biological behavior of pelvic floor support tissues. Among these, the culture of primary pelvic floor fibroblasts has become a core method for studying abnormalities in ECM metabolism. Multiple studies have shown that fibroblasts derived from the anterior vaginal wall of POP patients exhibit reduced collagen synthesis and enhanced MMP activity (128). For instance, comparative studies between POP patients and healthy controls have revealed that the TGF-β1 signaling pathway plays a critical role in regulating the imbalance in collagen I/III ratios (129). Additionally, stem cell culture offers novel perspectives for tissue regeneration. Specifically, endometrial-derived mesenchymal stem cells (SUSD2^+^ cells) demonstrate the potential to differentiate into fibroblasts and promote ECM deposition (130). These cellular models not only elucidate the molecular mechanisms of POP but also provide a foundation for drug screening platforms.

The introduction of mechanical loading systems has enabled the simulation of both physiological and pathological mechanical environments in pelvic floor tissues. For example, research teams designing uniaxial stretching systems have found that cyclic mechanical stress can induce morphological changes and cytoskeletal reorganization in fibroblasts (131). These biomechanical studies provide experimental evidence clarifying the association between birth trauma and POP pathogenesis, and help explain why multiparous women face a significantly elevated risk of prolapse (132).

The development of 3D culture and organoid models marks the entry of POP research into the era of microphysiological systems. Compared to traditional monolayer cultures, 3D models constructed using collagen-fibrin composite scaffolds better preserve the mechanosensory properties of cells (133). Breakthroughs have been made in building pre-vascularized tissue modules: researchers have successfully generated microspheres composed of human umbilical vein endothelial cells, fibroblasts, and adipose-derived stem cells using high-throughput techniques. These microspheres can spontaneously form capillary-like networks and, further through inosculation techniques, establish perfusable three-dimensional vascular networks within suspension systems or hydrogels. This lays the foundation for bioprinting large-scale vascularized tissues via a “bottom-up” approach (123). In the future, induced pluripotent stem cell (iPSC) technology holds promise for recombination with vaginal stromal cells to generate vaginal tissue mimics capable of responding to hormonal stimulation (134), opening new potential research avenues for studying the effects of estrogen and other hormones on vaginal epithelial cell differentiation and function *in vitro*. These advanced models not only overcome the limitations associated with interspecies differences in animal models but also accelerate drug discovery processes due to their high-throughput capabilities. For example, a recent study found that 17β-estradiol can delay the senescence of fibroblasts derived from POP patients by activating SIRT1 and inhibiting the p53/p21 signaling pathway (135).

A major challenge in translational research lies in integrating data from multi-scale models. Current limitations include phenotypic drift in primary cells after passaging and insufficient standardization of mechanical loading parameters, among others. Future efforts should focus on developing organoid-microfluidic coupled systems incorporating innervation, as well as creating “personalized disease models” based on patient-specific induced pluripotent stem cells (iPSCs). Notably, co-culture experiments using electrospun P4HB scaffolds and fibroblasts have demonstrated that material topology can guide oriented cell alignment (125), offering a new paradigm for biomaterial screening. With the application of single-cell sequencing technologies, researchers can now accurately identify POP‐related fibroblast subpopulations (136), which will advance the implementation of precision medicine strategies in pelvic floor reconstruction surgery.

### 6.2 Animal models

In the study of the pathogenesis of POP, animal models provide essential tools for understanding the roles of estrogen deficiency, mechanical injury, and genetic factors. The ovariectomy (OVX) model, which surgically removes estrogen sources to simulate the postmenopausal state, is a classical method for investigating the relationship between estrogen deficiency and POP. Using a rat model combining OVX and vaginal balloon dilation, Lin et al. identified changes in neurons and smooth muscle of the urethra and pelvic floor as a common pathway underlying stress urinary incontinence (SUI), demonstrating that this model effectively mimics the pathological features of human SUI (137). Similarly, Ferguson et al. confirmed that OVX rats exhibit reduced leak point pressure (LPP) following vaginal distension, indicating that estrogen levels directly influence the lower urinary tract’s response to injury (138). These findings are consistent with epidemiological data showing that postmenopausal estrogen decline exacerbates POP risk (1).

Mechanical injury models reveal the role of physical factors in POP by simulating birth trauma. Studies using vaginal balloon dilation have demonstrated that elastic fiber fragmentation and increased activity of matrix metalloproteinases (MMP-2 and MMP-9) are key mechanisms in POP (139). Further evidence shows that Fbln5 knockout mice fail to repair elastic fibers after vaginal distension, leading to irreversible POP, underscoring the central role of FBLN5 in tissue repair (97). Multiple studies indicate that the occurrence of POP results from the interaction between genetic susceptibility and environmental factors—such as birth injury. Among these, defects in genes including FBLN5 and LOXL1 may compromise the intrinsic strength of pelvic floor tissues, impairing their ability to repair and recover after mechanical insults like childbirth, thereby synergistically promoting the initiation and progression of POP (139).

Transgenic animal models provide precise tools for investigating specific gene functions in POP. HOXA11 knockout mice spontaneously develop POP due to developmental defects in the urethral support ligament (USL), accompanied by decreased expression of collagen III and TIMP1 (140). Tissue-specific knockout models of ERα or ERβ have elucidated the distinct roles of estrogen receptor subtypes: Orlicky et al. observed elevated expression of GPER and androgen receptor (AR) in POP phenotypes, while the contribution of ERα/β was less pronounced, suggesting that receptor subtypes may participate in the heterogeneous pathological processes of POP through different cell types, such as smooth muscle and endothelial cells (83). Additionally, LOXL1 knockout mice exhibit impaired elastic fiber assembly in pelvic support structures postpartum, consistent with reduced expression of LOX family genes in the ligaments of human POP patients. This highlights the conserved role of elastic metabolism in POP across species (15).

Spontaneous POP models, such as Fbln5/Loxl1-deficient mice, offer unique insights into genetic susceptibility. Molecular biological studies confirm that genetic defects (e.g., Fbln5−KO) are significant predisposing factors for POP. Approximately 92% of Fbln5-KO mice spontaneously develop POP by 6 months of age. After simulated birth injury via vaginal distension, their pelvic floor tissues exhibit sustained overexpression of cellular senescence markers (e.g., p53, γ-H2AX) and impaired repair capacity, revealing a novel mechanism underlying failed repair in pelvic support tissues (140). Such models closely recapitulate the genetic features of familial POP in humans, providing a foundation for investigating gene-environment interactions (141). It is noteworthy that these models exhibit complementarity: OVX models emphasize hormonal regulation, mechanical injury models highlight physical factors, while transgenic and spontaneous models elucidate molecular pathways. Together, they enable comprehensive simulation of the multifactorial etiology of POP (82). Future studies should integrate multi-model data to clarify the synergistic mechanisms involving estrogen signaling, ECM remodeling, and mechanical injury, thereby identifying targets for individualized therapy.

Although animal models, particularly rodents, have been indispensable in delineating specific etiological factors of POP—such as estrogen deficiency (OVX models), genetic susceptibility (Fbln5−KO, Loxl1−KO), and mechanical injury (vaginal distension) —they possess inherent translational limitations that necessitate critical consideration. A primary constraint stems from fundamental anatomical and biomechanical disparities (142, 143): the quadrupedal posture of rodents fails to replicate the lifelong gravitational and mechanical stress imposed on the bipedal human pelvic floor, limiting direct extrapolation of tissue response and pathophysiology. Secondly, these models typically lack the chronic, low-grade inflammatory microenvironment characterized by specific immune cell infiltration and cytokine profiles (67), which is increasingly recognized as a key driver of ECM degradation in human POP tissues. Furthermore, while invaluable for isolating molecular pathways, single-gene knockout models represent simplified, mono-factorial etiologies and cannot fully capture the multifactorial and progressive nature of human POP, which integrates genetic, hormonal, mechanical, and aging-related insults over decades. Future research should aim to develop models that better integrate these dimensions, such as through the use of non-human primates that spontaneously develop POP or by combining genetic susceptibility with chronic injury paradigms, to more accurately mirror the complex pathogenesis of the human condition (144).

### 6.3 Clinical study design

In investigating clinical study design for POP, the application of diverse research methods provides multidimensional evidence for understanding disease mechanisms and optimizing treatment strategies. Retrospective cohort studies are often used for initial exploration of risk factor-disease associations due to their efficiency and utilization of historical data. For example, a retrospective cohort study (2011–2021) from Taipei Veterans General Hospital analyzed electronic medical record data to compare the outcomes of robot-assisted sacrohysteropexy and sacrocolpopexy. The study found no significant differences between the two groups in operative time or most perioperative indicators but emphasized the importance of preoperative Pelvic Organ Quantification (POP-Q) assessment for surgical planning (145). Limitations of such studies include dependence on the completeness of historical records and potential unmeasured confounding factors. Prospective observational studies, on the other hand, allow more systematic collection of data linking specific exposures and outcomes. The Danish Nurse Cohort Study (1993–2015), which followed 11,114 nurses over the long term, identified BMI and parity as independent predictors of POP, while earlier menarche was associated with increased risk (146). Although this design reduces recall bias, caution is warranted regarding selection bias—for instance, the occupational characteristics of the nurse cohort may limit the generalizability of the findings.

Randomized controlled trials (RCTs), regarded as the gold standard in the hierarchy of evidence, are particularly suitable for evaluating the efficacy and safety of novel therapies. In the field of POP, RCT designs require careful attention to control group selection and blinding procedures. For example, a meta-analysis by Yu et al. (17), which included 7 RCTs with a total of 486 participants, demonstrated that local estrogen therapy (LET) significantly improved the vaginal maturation index (VMI) in postmenopausal women with POP (17). This suggests that while local estrogen may alleviate symptoms, it might not significantly alter anatomical support. Such studies often necessitate multicenter collaboration to ensure sufficient sample size and statistical power.

## 7 Challenges and future perspectives

### 7.1 Current challenges

The application of estrogen in the prevention and treatment of POP still faces numerous challenges and unresolved issues. A primary concern is the safety of long-term estrogen therapy, particularly the adverse effects associated with systemic administration, such as endometrial pathologies and thrombotic risks (107). Although local estrogen therapy is considered relatively safe, existing formulations have limited efficacy in repairing pelvic floor connective tissues that have already sustained severe structural damage. Clinical observations indicate that while local estrogen can improve vaginal epithelial thickness and collagen metabolism, its effect on anatomical restoration in patients with advanced POP is not significant (10, 112). This limitation may stem from irreversible extracellular matrix remodeling during POP progression, suggesting that estrogen monotherapy may miss the optimal therapeutic window (65).

A significant bottleneck in personalized therapy is the lack of reliable biomarkers for predicting treatment response. Studies have revealed considerable heterogeneity in the ERα/ERβ expression ratio among POP patients. Zbucka-Kretowska et al. demonstrated an elevated ERα/ERβ ratio in the pelvic floor tissues of these patients, a finding particularly pronounced in premenopausal individuals and characterized by increased ERα coupled with decreased ERβ expression. A similar trend was observed in postmenopausal cohorts (12). These dynamic alterations in receptor expression may account for the lack of response to estrogen therapy in some patients. Recently, Orlicky et al. proposed, via immunohistochemical analysis, that expression patterns of GPER and the androgen receptor (AR) might offer better discrimination of POP phenotypes than conventional ER subtypes, thereby providing a promising direction for novel predictive biomarkers (83). Nevertheless, large-scale prospective studies are still warranted to validate the clinical utility of these molecular markers.

A deeper scientific challenge lies in deciphering the interactions between estrogen signaling and the multifactorial pathogenic network of POP. Recent studies have revealed that estrogen receptors can mechanically activate the YAP/TAZ pathway, converting mechanical stimuli into changes in gene expression (93). This suggests that the mechanical microenvironment of pelvic floor tissues may remodel estrogen sensitivity. Conversely, local estrogen has been shown to delay cellular senescence through the SIRT1/p53 pathway (135). This complex cascade signaling network necessitates moving beyond single-pathway research paradigms and adopting integrated multi-omics analytical approaches.

Future research must achieve breakthroughs in three key dimensions: First, utilizing organoid and microfluidic chip technologies to establish pathological models of POP that simulate the combined effects of mechanical, hormonal, and senescent stress (140); second, conducting precision medicine trials based on molecular subtyping, such as testing specific agonists in subgroups with high ERβ expression; and finally, developing intelligent drug delivery systems to achieve spatiotemporally specific regulation within pelvic floor tissues. Only through such multidisciplinary collaborative efforts can we overcome the current limitations of fragmented knowledge and provide innovative strategies for the prevention and treatment of POP.

### 7.2 Future directions

The treatment of POP continues to face multiple challenges, particularly since the safety and efficacy of estrogen therapy have not been fully elucidated. Future research must achieve breakthroughs in several areas to develop more precise and personalized treatment regimens. A key direction involves targeted interventions against estrogen receptors (ERs). Current evidence suggests that an imbalance in the expression of ERα and ERβ in pelvic floor tissues may be implicated in the pathogenesis of POP (147). For instance, ERα expression is significantly reduced in the pelvic floor tissues of postmenopausal POP patients, while changes in ERβ expression remain controversial (1). Therefore, developing tissue-selective estrogen analogs or receptor modulators—such as selective ERβ agonists or non-genomic pathway modulators—may offer novel strategies for optimizing treatment. For example, studies have found that the expression of the GPER in pelvic smooth muscle and connective tissues correlates with POP phenotypes, indicating its potential as a therapeutic target (83). Additionally, the clinical effects of SERMs such as raloxifene are inconsistent: some studies suggest a reduced need for POP surgery (1), while others report an exacerbation of prolapse symptoms (107). Future efforts should focus on finer regulation of receptor subtypes to resolve these discrepancies.

Combination therapy represents another important research direction. The pathogenesis of POP involves multiple factors, including ECM degradation, oxidative stress, and chronic inflammation (65). Therefore, combining estrogen with modulators of other pathways may produce synergistic effects. For instance, while estrogen inhibits collagen degradation by downregulating MMPs (148), antifibrotic agents (e.g., TGF-β inhibitors) or antioxidants (such as N-acetylcysteine) could further enhance the protection of ECM (1). Furthermore, in the context of the low-estrogen environment in postmenopausal women, the combination of HRT with pelvic floor muscle training (PFMT) as an adjunct approach warrants exploration. However, the mechanisms underlying their potential synergy and long-term efficacy remain insufficiently supported by high-level clinical evidence, highlighting the need for further investigation (126).

Technological innovation is poised to revolutionize the treatment of POP. Optimizing local delivery systems is key to enhancing therapeutic efficacy. Currently available vaginal estrogen formulations (e.g., creams, rings), while effective in alleviating vaginal atrophy, exhibit limited structural restorative effects in POP (17). Novel sustained-release or targeted delivery systems—such as nanoparticles or hydrogels—may prolong drug residence time and reduce systemic side effects (122). For instance, a preliminary study demonstrated that a biodegradable scaffold loaded with 17β-estradiol promoted fibroblast proliferation and delayed cellular senescence (124). Furthermore, the integration of multi-omics technologies will advance precision medicine. Through transcriptomic, proteomic, and metabolomic analyses, researchers have identified key molecular markers associated with POP (e.g., LOXL1, FBLN5, TGF-β1). Future efforts combining these biomarkers with clinical data could enable molecular subtyping to guide individualized therapy. For example, the ERα rs2228480 polymorphism has been linked to POP risk (119), suggesting that genetic screening may help identify high-risk populations for early intervention.

Finally, advancing translational medicine urgently requires multidisciplinary collaboration. Advanced imaging techniques—such as three-dimensional ultrasound and magnetic resonance elastography—coupled with biomechanical assessments can provide objective indicators of treatment response (127). For example, dynamic MRI has been used to quantify defects in pelvic floor support structures (126), and such technologies may serve as tools for monitoring therapeutic efficacy in the future. The close integration of basic research and clinical practice is also essential. Existing contradictions, such as the heterogeneity in estrogen receptor expression, suggest that POP may comprise multiple subtypes (83), necessitating validation through large-scale cohort studies to clarify their clinical significance. In summary, future research should focus on integrating mechanistic exploration and technological application to ultimately achieve precision prevention and treatment of POP.

## 8 Conclusion

This review synthesizes current evidence to delineate the multidimensional role of estrogen signaling in pelvic floor connective tissue homeostasis and its dysregulation in POP pathogenesis. By integrating genomic and non-genomic actions mediated by ERα, ERβ, and GPER, we provide a cohesive framework that connects molecular mechanisms to tissue-level remodeling and clinical heterogeneity (56). The central contribution of this work lies in elucidating how disrupted estrogen signaling—characterized not merely by hormonal deficiency but more critically by an imbalanced ERα/ERβ ratio—orchestrates a pathological program of impaired ECM synthesis, accelerated degradation, and chronic inflammation, ultimately leading to support failure (55).

Current estrogen-based therapies, particularly local applications, offer limited and often transient benefits in advanced POP, highlighting a significant translational gap (57). To bridge this gap, future efforts must pivot towards personalized strategies. These include the development of receptor-subtype-specific agents (e.g., ERβ agonists, GPER modulators), biomarker-guided patient stratification utilizing multi−omics profiling, and the innovation of localized, sustained−delivery systems (53, 68). Advancing physiologically relevant research models and integrating biomechanical assessments will be crucial to fully decipher the estrogen−mechanics−aging interplay.

However, this review also acknowledges several limitations. Much of the current evidence is derived from *in vitro* cell lines or animal models, which may not fully recapitulate the complexity of human POP pathophysiology (57). The high clinical and molecular heterogeneity of POP suggests that dysregulation of a single pathway is unlikely to explain all cases, underscoring the need to explore broader pathophysiological networks. Future prospective clinical studies and large−scale, multi−center validation will be essential to translate mechanistic insights into effective, individualized therapies for POP.
